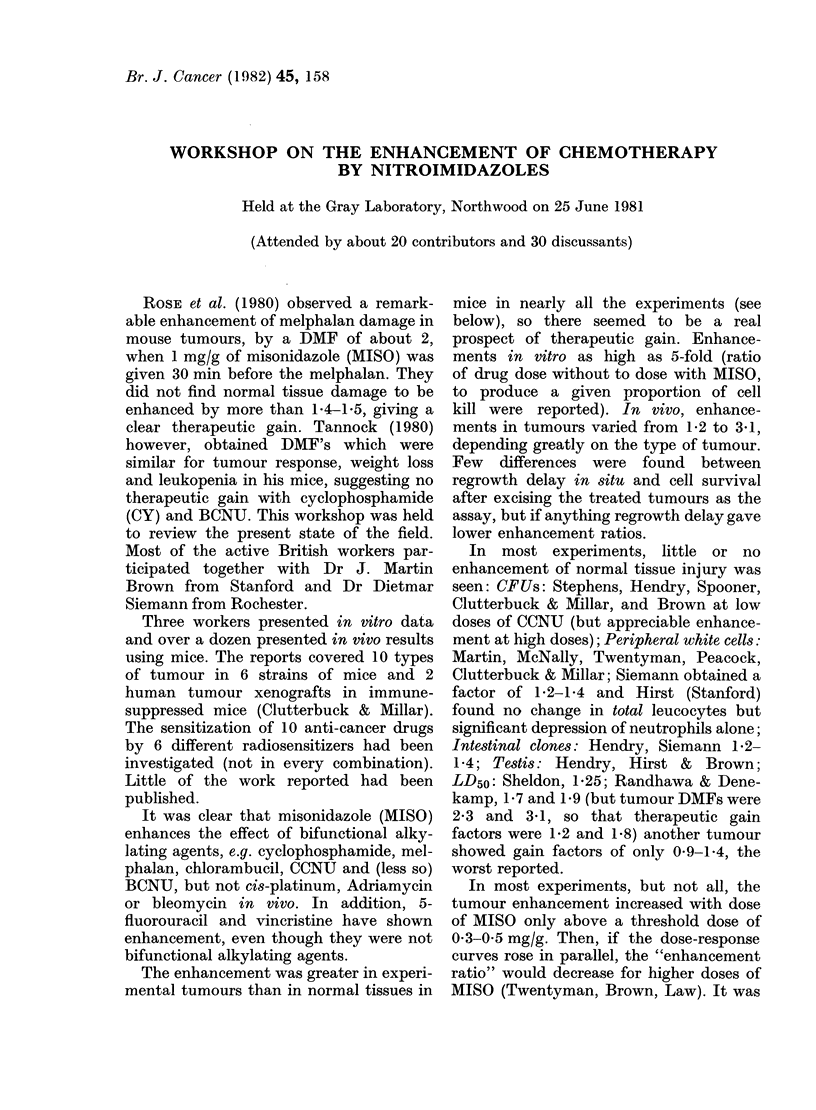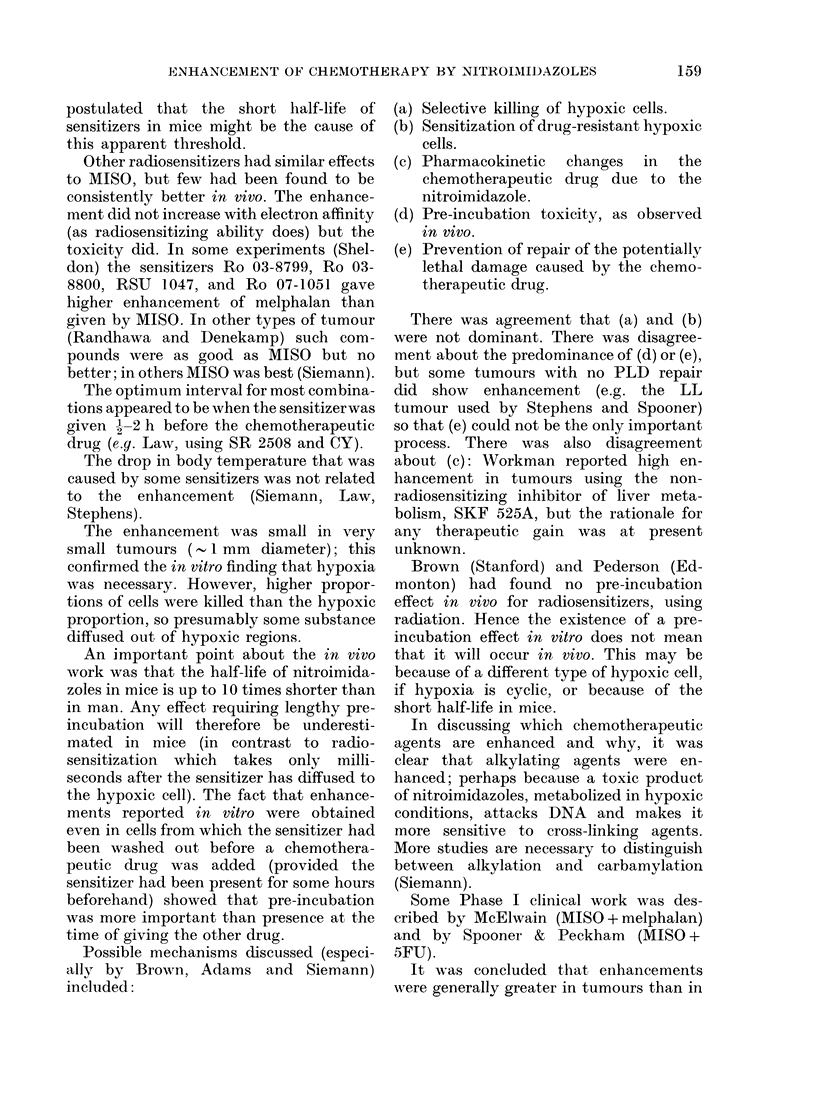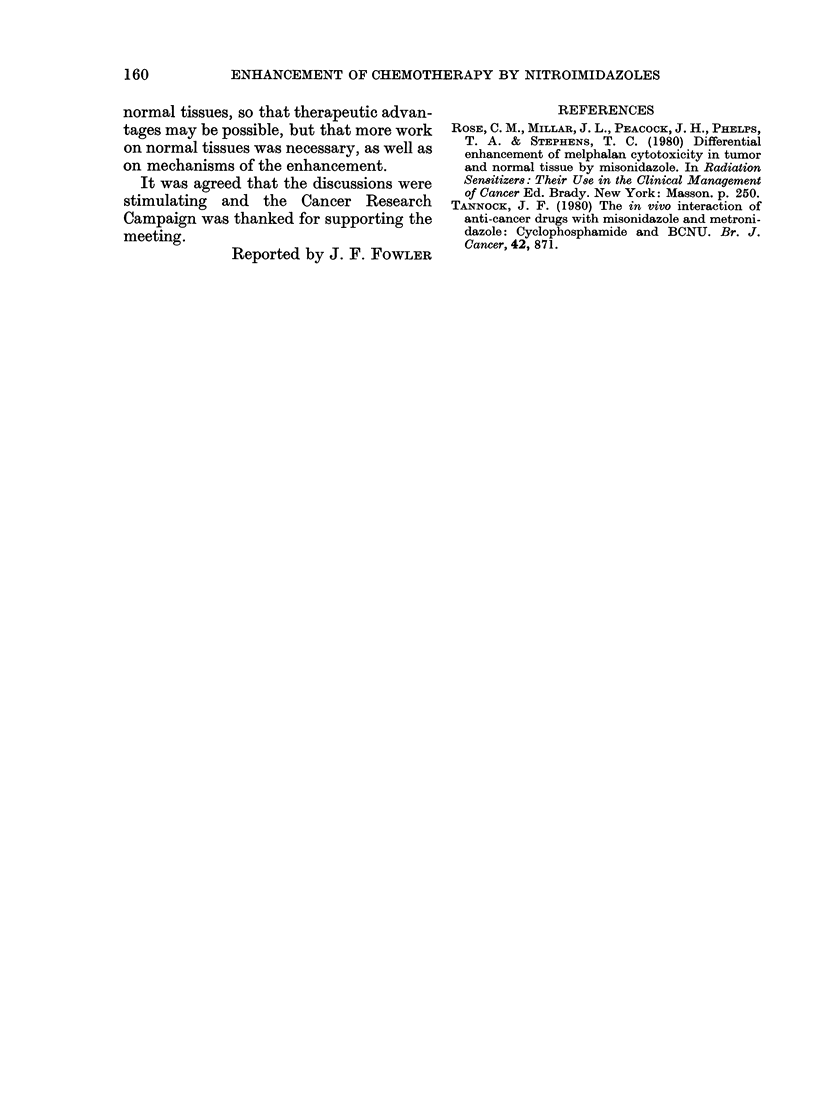# Proceedings of the Workshop on the Enhancement of Chemotherapy by Nitroimidazoles

**Published:** 1982-01

**Authors:** 


*Br. J. Cancer* (1982) **45**, 158

### WORKSHOP ON THE ENHANCEMENT OF CHEMOTHERAPY BY NITROIMIDAZOLES

Held at the Gray Laboratory, Northwood on 25 June 1981

(Attended by about 20 contributors and 30 discussants)

Rose *et al.* (1980) observed a remarkable enhancement of melphalan damage in mouse tumours, by a DMF of about 2, when 1 mg/g of misonidazole (MISO) was given 30 min before the melphalan. They did not find normal tissue damage to be enhanced by more than 1·4–1·5, giving a clear therapeutic gain. Tannock (1980) however, obtained DMF's which were similar for tumour response, weight loss and leukopenia in his mice, suggesting no therapeutic gain with cyclophosphamide (CY) and BCNU. This workshop was held to review the present state of the field. Most of the active British workers participated together with Dr J. Martin Brown from Stanford and Dr Dietmar Siemann from Rochester.

Three workers presented *in vitro* data and over a dozen presented *in vivo* results using mice. The reports covered 10 types of tumour in 6 strains of mice and 2 human tumour xenografts in immune-suppressed mice (Clutterbuck & Millar). The sensitization of 10 anti-cancer drugs by 6 different radiosensitizers had been investigated (not in every combination). Little of the work reported had been published.

It was clear that misonidazole (MISO) enhances the effect of bifunctional alkylating agents, *e.g.* cyclophosphamide, melphalan, chlorambucil, CCNU and (less so) BCNU, but not *cis*-platinum, Adriamycin or bleomycin *in vivo.* In addition, 5-fluorouracil and vincristine have shown enhancement, even though they were not bifunctional alkylating agents.

The enhancement was greater in experimental tumours than in normal tissues in mice in nearly all the experiments (see below), so there seemed to be a real prospect of therapeutic gain. Enhancements *in vitro* as high as 5-fold (ratio of drug dose without to dose with MISO, to produce a given proportion of cell kill were reported). *In vivo*, enhancements in tumours varied from 1·2 to 3·1, depending greatly on the type of tumour. Few differences were found between regrowth delay *in situ* and cell survival after excising the treated tumours as the assay, but if anything regrowth delay gave lower enhancement ratios.

In most experiments, little or no enhancement of normal tissue injury was seen: *CFU*s: Stephens, Hendry, Spooner, Clutterbuck & Millar, and Brown at low doses of CCNU (but appreciable enhancement at high doses); *Peripheral white cells:* Martin, McNally, Twentyman, Peacock, Clutterbuck & Millar; Siemann obtained a factor of 1·2–1·4 and Hirst (Stanford) found no change in *total* leucocytes but significant depression of neutrophils alone; *Intestinal clones:* Hendry, Siemann 1·2–1·4; *Testis:* Hendry, Hirst & Brown; $LD_{50}$: Sheldon, 1·25; Randhawa & Denekamp, 1·7 and 1·9 (but tumour DMFs were 2·3 and 3·1, so that therapeutic gain factors were 1·2 and 1·8) another tumour showed gain factors of only 0·9–1·4, the worst reported.

In most experiments, but not all, the tumour enhancement increased with dose of MISO only above a threshold dose of 0·3–0·5 mg/g. Then, if the dose-response curves rose in parallel, the "enhancement ratio" would decrease for higher doses of MISO (Twentyman, Brown, Law). It was postulated that the short half-life of sensitizers in mice might be the cause of this apparent threshold.

Other radiosensitizers had similar effects to MISO, but few had been found to be consistently better *in vivo*. The enhancement did not increase with electron affinity (as radiosensitizing ability does) but the toxicity did. In some experiments (Sheldon) the sensitizers Ro 03-8799, Ro 03-8800, RSU 1047, and Ro 07-1051 gave higher enhancement of melphalan than given by MISO. In other types of tumour (Randhawa and Denekamp) such compounds were as good as MISO but no better; in others MISO was best (Siemann).

The optimum interval for most combinations appeared to be when the sensitizer was given $\frac{1}{2}$–2 h before the chemotherapeutic drug (*e.g.* Law, using SR 2508 and CY).

The drop in body temperature that was caused by some sensitizers was not related to the enhancement (Siemann, Law, Stephens).

The enhancement was small in very small tumours ($\sim$1 mm diameter); this confirmed the *in vitro* finding that hypoxia was necessary. However, higher proportions of cells were killed than the hypoxic proportion, so presumably some substance diffused out of hypoxic regions.

An important point about the *in vivo* work was that the half-life of nitroimidazoles in mice is up to 10 times shorter than in man. Any effect requiring lengthy pre-incubation will therefore be underestimated in mice (in contrast to radiosensitization which takes only milliseconds after the sensitizer has diffused to the hypoxic cell). The fact that enhancements reported *in vitro* were obtained even in cells from which the sensitizer had been washed out before a chemotherapeutic drug was added (provided the sensitizer had been present for some hours beforehand) showed that pre-incubation was more important than presence at the time of giving the other drug.

Possible mechanisms discussed (especially by Brown, Adams and Siemann) included:

(a) Selective killing of hypoxic cells.
(b) Sensitization of drug-resistant hypoxic cells.
(c) Pharmacokinetic changes in the chemotherapeutic drug due to the nitroimidazole.
(d) Pre-incubation toxicity, as observed *in vivo*.
(e) Prevention of repair of the potentially lethal damage caused by the chemotherapeutic drug.

There was agreement that (a) and (b) were not dominant. There was disagreement about the predominance of (d) or (e), but some tumours with no PLD repair did show enhancement (e.g. the LL tumour used by Stephens and Spooner) so that (e) could not be the only important process. There was also disagreement about (c): Workman reported high enhancement in tumours using the non-radiosensitizing inhibitor of liver metabolism, SKF 525A, but the rationale for any therapeutic gain was at present unknown.

Brown (Stanford) and Pederson (Edmonton) had found no pre-incubation effect *in vivo* for radiosensitizers, using radiation. Hence the existence of a pre-incubation effect *in vitro* does not mean that it will occur *in vivo*. This may be because of a different type of hypoxic cell, if hypoxia is cyclic, or because of the short half-life in mice.

In discussing which chemotherapeutic agents are enhanced and why, it was clear that alkylating agents were enhanced; perhaps because a toxic product of nitroimidazoles, metabolized in hypoxic conditions, attacks DNA and makes it more sensitive to cross-linking agents. More studies are necessary to distinguish between alkylation and carbamylation (Siemann).

Some Phase I clinical work was described by McElwain (MISO + melphalan) and by Spooner & Peckham (MISO + 5FU).

It was concluded that enhancements were generally greater in tumours than in normal tissues, so that therapeutic advantages may be possible, but that more work on normal tissues was necessary, as well as on mechanisms of the enhancement.

It was agreed that the discussions were stimulating and the Cancer Research Campaign was thanked for supporting the meeting.

Reported by J. F. FOWLER

REFERENCES

ROSE, C. M., MILLAR, J. L., PEACOCK, J. H., PHELPS, T. A. & STEPHENS, T. C. (1980) Differential enhancement of melphalan cytotoxicity in tumor and normal tissue by misonidazole. In *Radiation Sensitizers: Their Use in the Clinical Management of Cancer* Ed. Brady. New York: Masson. p. 250.

TANNOCK, J. F. (1980) The *in vivo* interaction of anti-cancer drugs with misonidazole and metronidazole: Cyclophosphamide and BCNU. *Br. J. Cancer*, **42**, 871.